# New herbal composition (OA-F2) protects cartilage degeneration in a rat model of collagenase induced osteoarthritis

**DOI:** 10.1186/s12906-016-1535-9

**Published:** 2017-01-03

**Authors:** Pallavi S. Nirmal, Suresh D. Jagtap, Aarti N. Narkhede, Bhagyashri E. Nagarkar, Abhay M. Harsulkar

**Affiliations:** Department of Herbal Biotechnology, Interactive Research School for Health Affairs (IRSHA), Bharati Vidyapeeth Deemed University, Pune-Satara road, Pune, 411 043 Maharashtra India

**Keywords:** Formulation, Collagenase induced osteoarthritis, Cartilage, Synovium

## Abstract

**Background:**

Prevalence of osteoarthritis (OA) is on rise on the global scale. At present there are no satisfactory pharmacological agents for treating OA. Our previous study showed that *Sida cordifolia* L. and *Zingiber officinale* Rosc. had protective effect on cartilage. Here, we describe the effect of OA-F2, a herbal formulation prepared using combination of these two plants in alleviating OA associated symptoms in a rat model of collagenase-induced OA.

**Methods:**

OA was induced by intra-articular injection of collagenase type II in wistar rats. Diclofenac (10 mg/kg) was used as a reference control. Rats (*n* = 6) were divided into 6 groups: Healthy control (HC), osteoarthritic control (OAC), diclofenac (DICLO), OA-F2L (135 mg/kg), OA-F2M (270 mg/kg) and OA-F2H (540 mg/kg). The effects of the 20 days treatment were monitored by parameters like knee diameter, paw volume, paw retraction; serum C-reactive protein (CRP), alkaline phosphatase (ALP) and glycosaminoglycan (GAG). Radiography and histopathology of knee joint were also studied. Additionally, gene expression was studied from isolated synovium tissue proving anti-osteoarthritic potential of OA-F2.

**Results:**

Oral administration of OA-F2 has significantly prevented knee swelling compared to OAC; OA-F2 and DICLO, significantly reduced paw volume compared to OAC. Paw latency was remarkably increased by OA-F2 compared to OAC. OA-F2L (−0.670, *p* < 0.001), M (−0.110, *p* < 0.05) and H (0.073) has markedly reduced levels of CRP compared to DICLO. OA-F2L (*p* < 0.05), M (*p* < 0.001) and H (*p* < 0.05) significantly reduced ALP levels, compared to DICLO. GAG release in the serum was also significantly lowered in OA-F2 treated group compared to DICLO. Radiological and histopathological observations showed cartilage protection by OA-F2. OA-F2 has upregulated SOD and GPx. Upregulated CAT expression was observed in OA-F2M and H. Considerable down-regulation of expression of MMP-3 and MMP-9 was observed in all the groups. Up-regulation of TIMP-1 was observed in rats treated with OA-F2L, H and DICLO.

**Conclusion:**

OA-F2 has shown therapeutic effects in rat model of collagenase induced OA by demonstrating cartilage protection through controlling MMPs and improving anti-oxidant levels in arthritic synovium and is a potent candidate for further drug development and treatment for OA.

## Background

Osteoarthritis (OA) is a complex multifactorial disease of whole joint [1]. Pathological loss of cartilage reflects the imbalance between catabolic and anabolic mechanism of cartilage remodeling, which is influenced by oxidative and inflammatory changes in the surrounding tissues especially synovium and subchondral bone. The prevalence of OA in Indian population is estimated to be 22–39%; it is a major cause of joint pain and disability in aging population imposing a high social burden [2–4]. However, mechanism involved behind this degenerative disease is poorly understood that reflects in the current lack of effective medical therapies [5].

The cascade starting from reactive oxygen species (ROS) leading to inflammation and increased matrix metalloproteinases are the important factors of degradation of matrix components [6]. They have been found to be overproduced in OA cartilage and synovium [6]. Previous reports have shown that, ROS scavengers can slow-down cartilage loss [6]. ROS contribute to cartilage degradation by sustaining the activity of catabolic cytokines and by reducing cartilage repair capacities. When ROS production exceeds the antioxidant capacities of the cell, an “oxidative stress” occurs that contributes to structural and functional cartilage damages. Oxidative stress is found to be elevated in cartilage and synovial tissues, which contributes to disease progression by increasing production of inflammatory mediators and matrix degrading enzymes [7]. To prevent toxicity by ROS, chondrocytes express a well co-ordinated anti-oxidant enzyme system in the form of SOD, CAT and GPX [6]. Matrix metalloproteinases (MMPs), which contribute to the accelerated degradation of extracellular matrix and tissue inhibitors of metalloproteinases (TIMPs) that regulates the MMP activity, play an important function in cartilage matrix turnover [8]. However, in OA affected joint tissues, up-regulation of MMPs is reported higher compared to TIMPs [8]. Altogether, these observations support the concept of antioxidant therapy along with modulation of MMP-TIMP might also decrease the progression of OA.

Unfortunately, at present there is no medicine or therapy that can target cartilage protection. Consequently, there is a growing interest in complementary and alternative medicines (CAM) either in the form of nutraceuticals or in the form of herbals that primarily thought to be chondroprotective and may even repair cartilage. It is therefore interesting to explore traditional systems of medicine for a potential drug that may help to reduce inflammation, protect cartilage damage, improve joint functions and restore patient’s activity levels.

In our earlier studies, anti-osteoarthritic potential of six herbs was shown, which are commonly used by Ayurvedic physicians for the treatment of OA viz. *Sida cordifolia* L., *Piper longum* L., *Zingiber officinale* Rosc., *Ricinus communis* L., *Vitex negundo* L. and *Tribulus terrestris* L., in collagenase type II induced OA (CIOA) rat model [9]. *Sida cordifolia* L. has been proven to be effective in balancing *Vata dosha*, which is one of the basic principals in *Ayurveda* [10]. *S. cordifolia* is shown to inhibit cyclooxygenase (COX) leading to the inhibition of prostaglandin (PGE) synthesis [11]. *Zingiber officinale* Rosc. is a common spice with several ethnomedicinal and nutritional values. It has been used traditionally for treating arthritic conditions [12]. It decreases interleukin-1 (IL1), nitric oxide (NO) and PGE2 and inhibit leukotriene B4 (LTB4) production in osteoarthritic cartilage [11, 13], it also inhibit production of tumor necrosis factor-α (TNF-α) in human osteoarthritic synoviocytes and chondrocytes [14]. Srivastsava et al. [15] reported 6-gingerol, active compound from its rhizome inhibit nuclear factor kappa B (NF-κB), activator protein-1 (AP-1), TNF-α, interleukin 12 (IL12), inducible NO synthase and COX-2 [16]. *Z. officinale* inhibits biotransformation of arachidonic acid into inflammatory prostaglandin. In a clinical study on knee OA, using formulation containing *Z. officinale* as one of its ingredients, there was significant reduction in pain score [17].

In the present study, a novel herbal formulation named as OA-F2 was designed comprising a proportionate combination of *S. cordifolia* and *Z. officinale*, these herbs have shown appreciable anti-osteoarthritic potential in our previous studies [9]. We have studied efficacy of oral administration of OA-F2 in alleviating OA associated symptoms by using collagenase-induced OA (CIOA) in rats and its underlying molecular mechanism.

## Methods

### Individual material

Roots of *S. cordifolia* and rhizomes of *Z. officinale* were obtained from Green Pharmacy (Pune, Maharashtra, India) in dried form. The drugs were then identified and authenticated in house by *Ayurvedic* experts using API parameters viz. total ash, acid insoluble ash, alcohol soluble extractive, water soluble extractive and pH of extract.

### The composition

Roots of *S. cordifolia* and rhizomes of *Z. officinale* were obtained, they were shade dried. The composition material of OA-F2 was prepared by blending powders of *S. cordifolia* roots and *Z. officinale* rhizomes, with 1 : 1 ratio and given in their powder form after sieving through mesh No.120.

### Experimental animals

Institutional animal ethics committee approval for the experimental protocol was obtained from Bharati Vidyapeeth Deemed University, Medical College, Pune, before initiation of the study (BVDUMC/CPCSEA/2679/2012-13). Female wistar rats (*n* = 36) weighing between 180 and 300 gm were used for the experiment. They were housed for two weeks in solid bottomed polypropylene cages for acclimatization before use, maintained under standard conditions and fed standard rat chaw with water *ad libitum*. Guidelines laid down by the CPCSEA were observed throughout the study for animal handling and experimentation.

### Treatment

Rats were obtained from National Institute of Biosciences (Pune, India) and divided into 6 groups of 6 animals each. Group I (HC) animals served as a control (Received normal sterile saline injection 50 μl intra-articularly to the right knee). All other groups were injected 50 unit of collagenase type II intra-articularly to right knee. Group II (OAC) was maintained as osteoarthritic control and were not given any treatment, Group III (DICLO) was positive control, given standard drug diclofenac (10 mg/kg *p.o*); Group IV (OA-F2L) served with test drug OA-F2 at the dose of 135 mg/kg b. wt., Group V (OA-F2M) served with test drug OA-F2 at the dose of 270 mg/kg b. wt., Group VI (OA-F2H) served with test drug OA-F2 at the dose of 540 mg/kg b. wt.

### Collagenase type II-induced osteoarthritis (CIOA)

Rats were anesthetized with diethyl ether (Merck, India). Right knee joints were shaved. Group I were injected with 50 μl of normal saline solution and served as control. Animals from the second group were intra-articularly injected with 50 μl collagenase type II (Sigma Aldrich, USA) dissolved in saline (50 unit) into the right knee joint [9, 18]. The injection was performed on 1st and 4th day of the experiment [19]. Standard drug diclofenac (Manufactured by Medreich Limited, India) was given at the dose of 10 mg/kg b. wt. in 0.5% sodium-carboxy methyl cellulose, based on previous reports [20]. The formulation doses were selected based on the human dose mentioned in the *Ayurvedic* literature and was calculated for animal use based on the body surface area ratio [21]. Doses were administered as oral suspension in water, once per day by feeding needles from day 14 to 34.

### Effect of OA-F2 on Knee diameter, Paw volume and Paw latency in CIOA rats

Knee diameter was measured on 0th, 5th, 10th, 15th, 20th, 25th and 30th day using digital vernier caliper (Mitutoyo, Japan), mean changes in joint swelling after treatment were calculated. Paw volume was measured once in a week using digital plethysmometer (Orchid Scientifics, India) and % Inhibition of paw edema with respect to OAC group was calculated using following formula: Percent inhibition of paw edema = (Vc-Vt)/Vc*100, Where Vc: Paw Volume of OAC group, Vt: Paw Volume of test group. Paw latency measured before and after treatment using tail flick unit (Ugobasile unit, Italy), Paw latency after treatment was determined.

### Determination of markers from serum

Blood was removed before and after treatment through retro-orbital vein puncture and serum was separated. C-reactive protein (CRP) was determined using quantitative turbidometric test. Alkaline phosphatase (ALP) activity was determined using Mod. Kind and King’s method (Coral clinical systems, Tulip group, India). Glycosaminoglycans (GAG) assay was performed as described in Hoemann et al. [22].

### Radiological analysis

Before the termination of experiment, the animals were anesthetized using diethyl ether and anterioposterior (AP) X-rays using (GE Medical Systems- DXD 300, 300MA, America) thermal laser AGFA digital photo films (digitizer CR-30, AGFA photo film, Belgium) were taken for the knee joints of the animals to evaluate the cartilage degradation and joint space reduction. The X-ray was operated at 300 MA, 50 KV, 0.02 s exposure time and a 100 cm tube to film distance for AP projection.

### Histopathological analysis

On day 34th, animals were sacrificed, the right knee joint was dissected and fixed in 10% phosphate buffered formalin and were decalcified, sectioned and finally stained with hematoxyline and eosin (H and E), safranin-O and masson’s trichome. Synovium was dissected out and fixed in 10% phosphate buffered formalin for staining. Tissue samples were prepared for light microscopy using standard procedures.

### Quantitative real-time reverse transcription-polymerase chain reaction (qPCR) analysis

Animals were sacrificed at the end of the experiment and their synovial tissue was removed, flash frozen immediately in liquid nitrogen, stored at −80 °C and subsequently used for quantitative real-time reverse transcription-polymerase chain reaction (qPCR) studies. Total RNA from isolated knee synovium tissue was extracted using TRIZOL reagent (Sigma Aldrich, USA) with PureLink RNA mini kit (Invitrogen CA, USA). Quality of the isolated RNA was determined using denaturing agarose gel electrophoresis followed by quantification by measuring absorbance at 260 nm. The first strand cDNA was synthesized from 1 μg of total RNA using the SuperScript first-strand synthesis system for quantitative real-time PCR (Applied Biosystems CA, USA). qPCR analysis was performed with the help of a StepOne realtime PCR system (Applied Biosystems, CA, USA) using TaqMan gene expression assays (Applied Biosystems, CA, USA). Taqman gene expression master mix was procured from Applied Biosystems (CA, USA). Cycling conditions were 50 °C for 2 min; 95 °C for 10 min; and 40 cycles of 95 °C for 15 s, 60 °C for 1 min. The Taqman gene expression assays that were used in this study are SOD (Sod1; Rn00566938_m1), GPx (Gpx1; Rn00577994_g1), CAT (Cat; Rn00560930_m1), MMP-3 (Mmp3; Rn00591740_m1), MMP-9 (Mmp9 Rn00579162_m1) and TIMP-1 (Timp1; 1 Rn00587558_m1). The data was analyzed using Data Assist software version 3.0. The data is representative of synovium from at least three animals. The relative abundance of the RNA was calculated to the amount of β-actin (Actb; Rn00667869_m1) using StepOne software version 2.2.2, DataAssist version 3.0 (Applied Biosystems, CA, USA) and the ΔΔ Ct method [23].

### Statistical analysis

Statistical analysis was carried out by one-way analysis of variance (ANOVA) followed by Dunnett’s multiple comparison test using GraphPad Prism software version 5.0 (GraphPad Software Inc., CA, USA). The data were expressed as Mean ± Standard Error (SE).

## Results

### Effect of OA-F2 on Knee diameter, Paw volume and Paw latency in CIOA rats

**Fig. 1:**
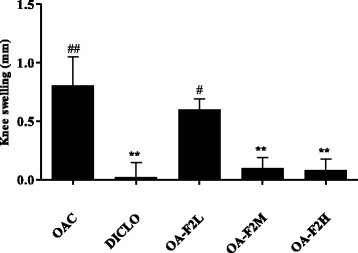
Effect of OA-F2 on knee swelling. Values are expressed as Mean ± SE; *n* = 6. ^**^ *p* < 0.01 compared to OAC. ^#^ *p* < 0.05, ^##^ *p* < 0.01 compared to DICLO. Data were analyzed by One-Way ANOVA followed by the Dunnett’s multiple comparison test. OAC: Osteoarthritic control, DICLO: Positive control, OA-F2L: OA-F2 at 135 mg/kg b. wt., OA-F2M: OA-F2 at 270 mg/kg b. wt., OA-F2H: OA-F2 at 540 mg/kg b. wt

**Fig. 2:**
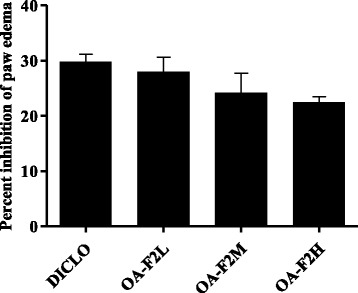
Effect of OA-F2 on percent inhibition of paw edema. Values are expressed as Mean ± SE; *n* = 6. DICLO: Positive control, OA-F2L: OA-F2 at 135 mg/kg b. wt., OA-F2M: OA-F2 at 270 mg/kg b. wt., OA-F2H: OA-F2 at 540 mg/kg b. wt

**Fig. 3:**
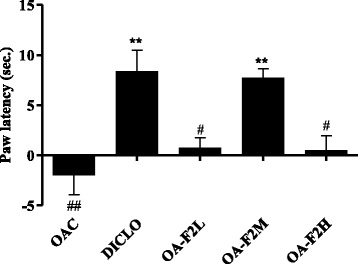
Effect of OA-F2 on paw latency. Values are presented in Mean ± SE; *n* = 6. ^**^ *p* < 0.01 compared to OAC. ^#^ *p* < 0.05, ^##^ *p* < 0.01 compared to DICLO. Data were analyzed by One-Way ANOVA followed by Dunnett’s multiple comparison test. OAC: Osteoarthritic control, DICLO: Positive control, OA-F2L: OA-F2 at 135 mg/kg b. wt., OA-F2M: OA-F2 at 270 mg/kg b. wt., OA-F2H: OA-F2 at 540 mg/kg b. wt

OAC group demonstrated a significant increase in joint diameter (*p* < 0.01) (Fig. 1). Both DICLO (*p* < 0.01) and OA-F2 treated group substantially attenuated the increase in joint diameter. Treatment with OA-F2L, M and H showed 25%, 88% (*p* < 0.01) and 89% (*p* < 0.01) reduction in joint swelling, respectively (Fig. 1). DICLO produced considerable inhibition in the paw edema by 29.83 ± 1.33% (Fig. 2). Groups OA-F2L, M and H produced 28.02 ± 2.58%, 24.19 ± 3.50% and 22.48 ± 1.01% inhibition of paw edema, respectively (Fig. 2), which shows OA-F2 has anti-inflammatory effect. OA-F2L, OA-F2M (*p* < 0.01), OA-F2H and DICLO (*p* < 0.01) showed considerable increase in right paw withdrawal latency compared to OAC (Fig. 3).

### Effect of OA-F2 on serum CRP, ALP and GAG levels in CIOA rats

**Fig. 4:**
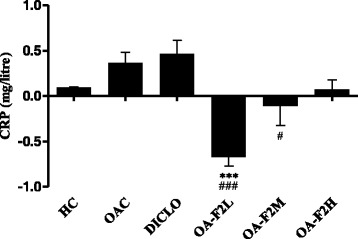
Effect of OA-F2 on CRP levels in the serum. Values are expressed as difference in Mean ± SE between before (13th day) and after (34th day) treatment; *n* = 6. ^***^ *p* < 0.001 compared to OAC. ^#^ *p* < 0.05, ^###^ *p* < 0.001 compared to DICLO. Data were analyzed by One-Way ANOVA followed by Dunnett's multiple comparison test. HC: Healthy control, OAC: Osteoarthritic control, DICLO: Positive control, OA-F2L: OA-F2 at 135 mg/kg b. wt., OA-F2M: OA-F2 at 270 mg/kg b. wt., OA-F2H: OA-F2 at 540 mg/kg b. wt

**Fig. 5:**
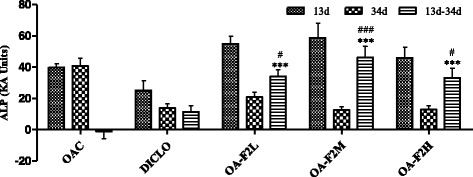
Effect of OA-F2 on ALP levels in the serum. Values are expressed as Mean ± SE; *n* = 6. ^***^ *p* < 0.001 compared to OAC. ^#^ *p* < 0.05, ^###^ *p* < 0.001 compared to DICLO. Data were analyzed by One-Way ANOVA followed by Dunnett’s multiple comparison test. OAC: Osteoarthritic control, DICLO: Positive control, OA-F2L: OA-F2 at 135 mg/kg b. wt., OA-F2M: OA-F2 at 270 mg/kg b. wt., OA-F2H: OA-F2 at 540 mg/kg b. wt

**Fig. 6:**
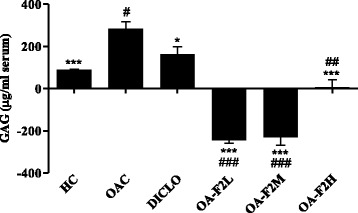
Effect of OA-F2 on GAG release in the serum. Values are expressed as difference in Mean ± SE between before (13th day) and after (34th day) treatment; *n* = 6. ^*^ *p* < 0.05; ^***^ *p* < 0.001 compared to OAC. ^#^ *p* < 0.05; ^##^ *p* < 0.01; ^###^ *p* < 0.001 compared to DICLO. Data were analyzed by One-Way ANOVA followed by Dunnett’s multiple comparison test. HC: Healthy control, OAC: Osteoarthritic control, DICLO: Positive control, OA-F2L: OA-F2 at 135 mg/kg b. wt., OA-F2M: OA-F2 at 270 mg/kg b. wt., OA-F2H: OA-F2 at 540 mg/kg b. wt

As a result of inflammation induced by collagenase injection, the levels of CRP, ALP and GAG were increased in all OA control rats. Elevated levels of CRP in OA have been correlated with disease progression [24]. Serum CRP levels were significantly decreased by OA-F2L (*p* < 0.001) and OA-F2M compared to OAC (Fig. 4). In OA, ALP levels increases in the serum [25]. OA-F2L, M and H significantly (*p* < 0.001) decreased ALP compared to OAC (Fig. 5). Proteoglycans consist of a protein core with GAG side chains. During inflammation and cartilage degradation, GAG monomers are released, which can be detected in synovial fluid and even in blood serum serving as the clinical parameter of OA [26]. Serum GAG levels were significantly increased in OAC group compared to HC (*p* < 0.001) (Fig. 6). DICLO (*p* < 0.05), OA-F2L (*p* < 0.001), OA-F2M (*p* < 0.001) and OA-F2H (*p* < 0.001) have significantly decreased GAG levels compared to OAC (Fig. 6).

### Radiography and Histopathology evaluation of rat knee joint

**Fig. 7:**
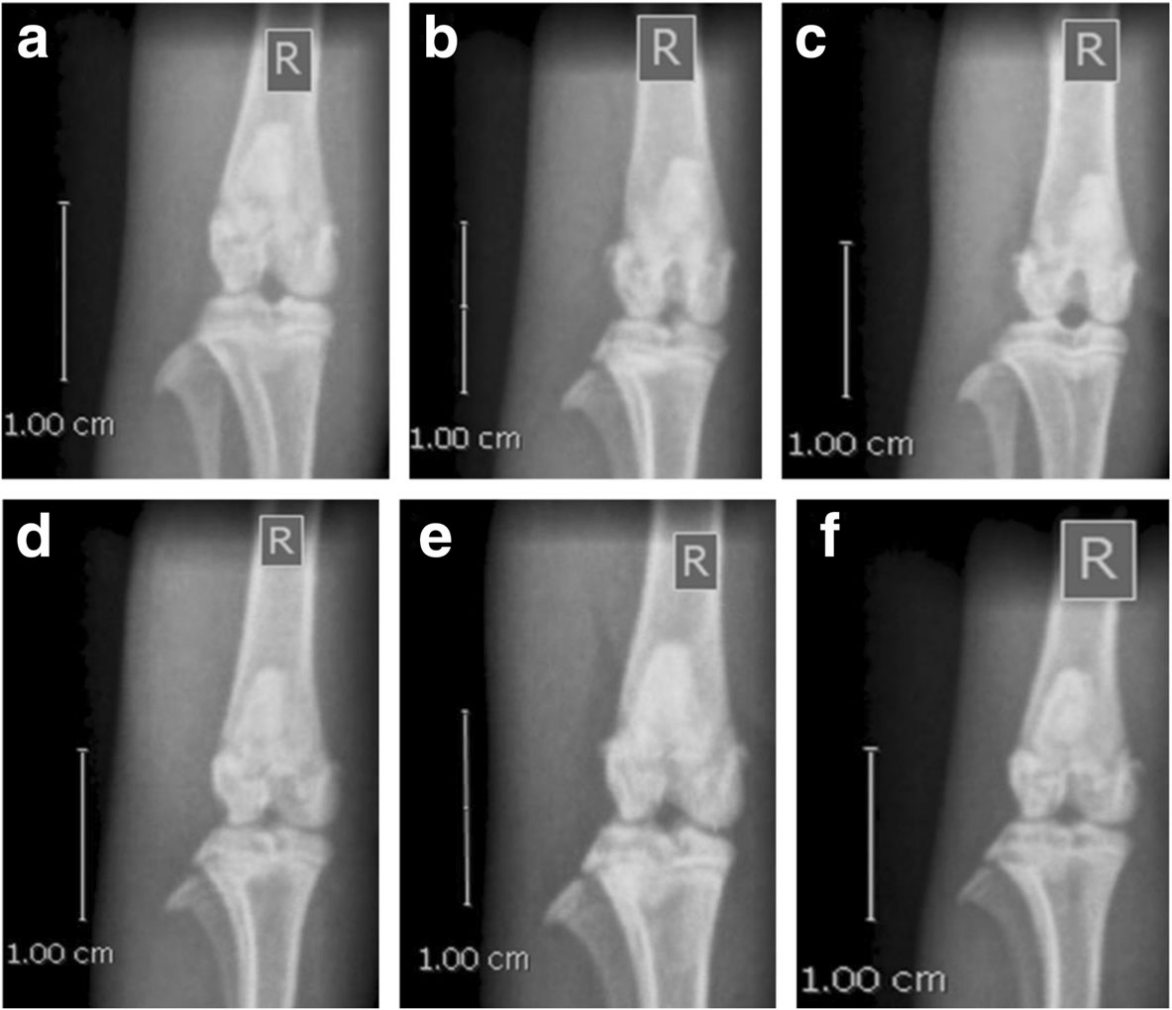
Right knee joint radiographs (in the Anterior Posterior view) of control and experimental animals. All radiographs were taken keeping object to x-ray source distance constant (100 cm) and scale is showing extent of zoom. **a** Saline injected groups showed smooth articular surface (arrow) while in (**b**) OAC and (**c**) DICLO group showed severe changes of OA; Comparatively (**d**) OA-F2L, (**e**) OA-F2M and (**f**) OA-F2H group showed minimal changes of OA

Radiographic images of the joints were compared between the right knee (induced) and left knee for erosion of articular cartilage, reduced joint space and osteophyte formation. The induced knee in OAC group showed severe osteoarthritic changes, such as marked erosion of articular cartilage and reduction in joint space due to loss of articular cartilage (Fig. 7b). In HC group, the patello-femoral joints of the knee appeared normal without obvious abnormalities in surrounding tissue (Fig. 7a). DICLO treated group showed severe changes of OA (Fig. 7c). In the OA-F2L group, joint space was found protected with no signs of erosions at articular surface (Fig. 7d). Regression in erosions of articular surface was also seen in rats treated with OA-F2M (Fig. 7e) and joint space in the knee joint was improved after treatment with OA-F2H (Fig. 7f). Thus, OA-F2 appeared to possess cartilage protection abilities.

**Fig. 8:**
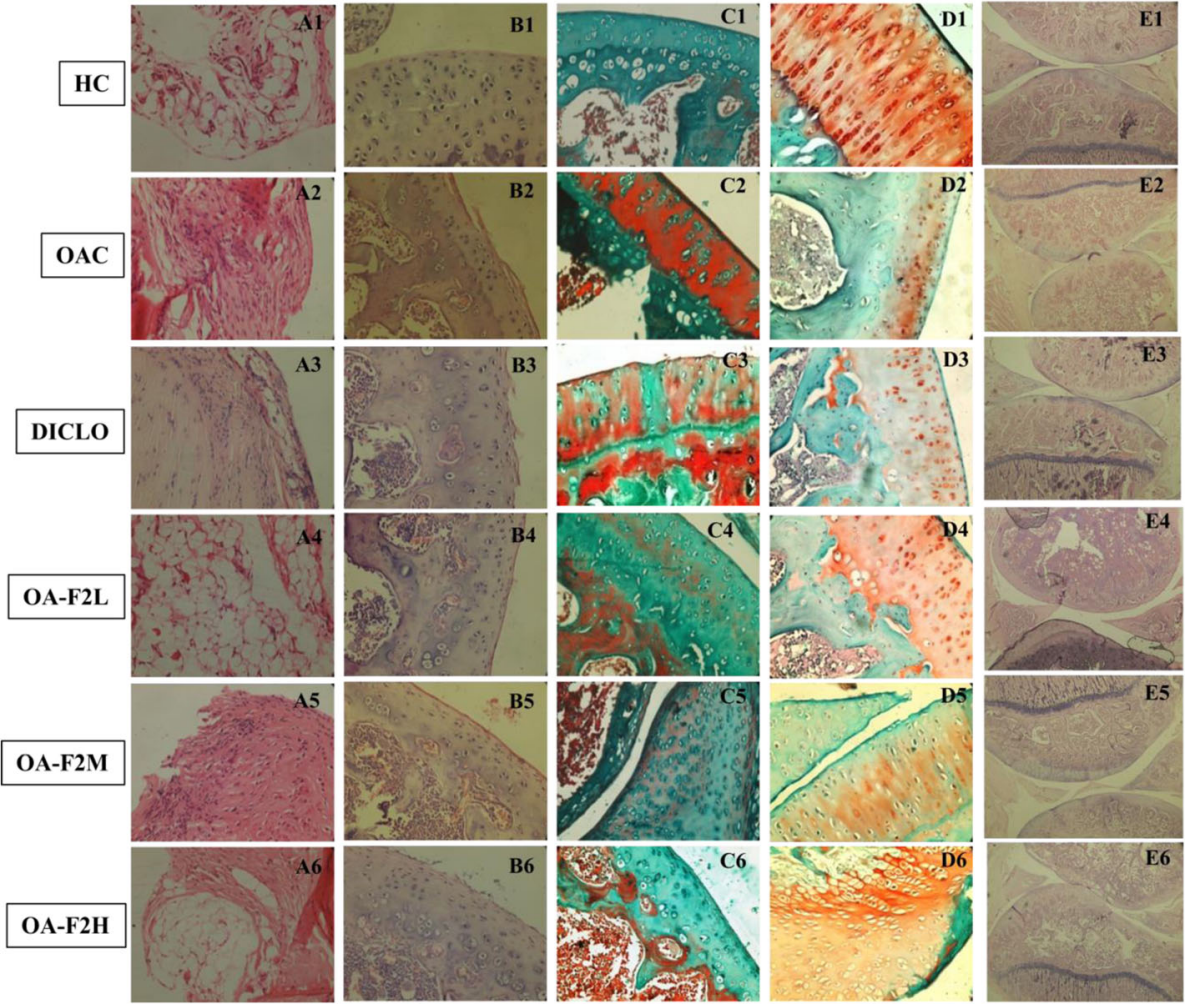
Histopathology pictures of synovial membrane and cartilage. First column (**a**1-6) showing photographs of synovium stained with H and E and further are photographs of cartilage stained with H and E (**b**1-6), Masson’s trichome (**c**1-6) and safranin-O (**d**1-6) respectively. Last column (**e**1-6) showing photographs of whole knee joint stained with H and E. HC: Healthy control, OAC: Osteoarthritic control, DICLO: Positive control, OA-F2L: OA-F2 at 135 mg/kg b. wt., OA-F2M: OA-F2 at 270 mg/kg b. wt., OA-F2H: OA-F2 at 540 mg/kg b. wt., H and E: hematoxyline and eosin

Histology of the synovium in HC group showed 1–2 layers of cells without cellular infiltration (Fig. 8a1). In OAC group, lining cells showed mild increase in layer and stroma showed mononuclear infiltrate (Fig. 8a2). Compared to DICLO, OA-F2L and OA-F2H have shown protection to synovium. Histology of the articular cartilage in HC group showed cartilage with normal volume, smooth resilient surface with all distinguishable zones intact (Fig. 8b1). Chondrocyte pathology was within normal limits (Fig. 8c1) and the matrix was densely stained red with Safranin (Fig. 8d1). Intra-articular injection of collagenase produced erosion of cartilage with irregular surface and fibrillation, chondrocytes were seen in clusters in joint tissues of OAC and DICLO (Fig. 8c2, c3). As compared with control group (HC), proteoglycans (PGs) integrity was found decreased in the upper cartilage zone (Fig. 8d2, d3). Comparatively, treatment with OA-F2L and M has prevented cartilage degradation. OA-F2 also has shown more collagen deposition with reduced cleft formation compared to OAC and DICLO (Fig. 8c4, c6). PGs layer density was also increased after the OA-F2 treatment, compared to OAC and DICLO (Fig. 8d4, d6). Thus, OA-F2 protected chondroid matrix as well as prevented cartilage surface degeneration. Figure 8e1 to e6 (H and E staining) gives an overview of the whole joint at low magnification.

### Effect of OA-F2 on expression of anti-oxidant genes, MMPs and TIMP in CIOA rats

**Fig. 9:**
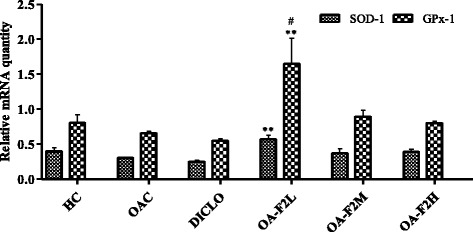
Effect of OA-F2 on the mRNA expression of SOD-1 and GPx-1 in the synovium of rats. Values are expressed as Mean ± SE (*n* = 3). Comparisons were done between OAC/HC and each individual treated group by Dunnett’s multiple comparison test. **p < 0.01 Compared to OAC. ^#^ *p* < 0.05 compared to HC. HC: Healthy control, OAC: Osteoarthritic control, DICLO: Positive control, OA-F2L: OA-F2 at 135 mg/kg b. wt., OA-F2M: OA-F2 at 270 mg/kg b. wt., OA-F2H: OA-F2 at 540 mg/kg b. wt

**Fig. 10:**
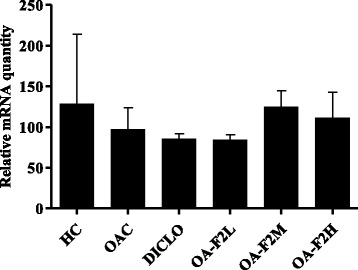
Effect of OA-F2 on the mRNA expression of CAT in the synovium of rats. Values are expressed as Mean ± SE (*n* = 3). HC: Healthy control, OAC: Osteoarthritic control, DICLO: Positive control, OA-F2L: OA-F2 at 135 mg/kg b. wt., OA-F2M: OA-F2 at 270 mg/kg b. wt., OA-F2H: OA-F2 at 540 mg/kg b. wt

**Fig. 11:**
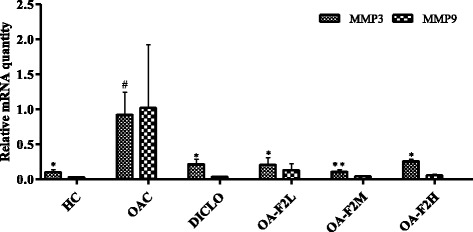
Effect of OA-F2 on the mRNA expression of MMP3 and MMP9 in the synovium of rats. Values are expressed as Mean ± SE (*n* = 3). Comparisons were done between OAC/HC and each individual treated group by Dunnett’s multiple comparison test. ^*^ *p* < 0.05; ^**^ *p* < 0.01 Compared to OAC. ^#^ *p* < 0.05 compared to HC. HC: Healthy control, OAC: Osteoarthritic control, DICLO: Positive control, OA-F2L: OA-F2 at 135 mg/kg b. wt., OA-F2M: OA-F2 at 270 mg/kg b. wt., OA-F2H: OA-F2 at 540 mg/kg b. wt

**Fig. 12:**
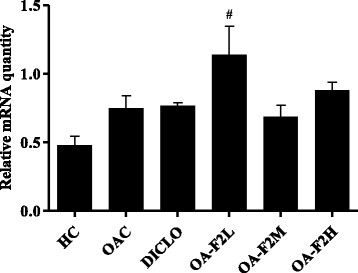
Effect of OA-F2 on the mRNA expression of TIMP1 in the synovium of rats. Values are expressed as Mean ± SE (*n* = 3). Comparisons were done between OAC/HC and each individual treated group by Dunnett’s multiple comparison test. ^#^ *p* < 0.05 compared to HC. HC: Healthy control, OAC: Osteoarthritic control, DICLO: Positive control, OA-F2L: OA-F2 at 135 mg/kg b. wt., OA-F2M: OA-F2 at 270 mg/kg b. wt., OA-F2H: OA-F2 at 540 mg/kg b. wt

Osteoarthritic control rats showed down-regulation of SOD, GPx and CAT in the knee joint synovium by ~1.22-fold, ~1.22-fold and ~1.33-fold, respectively compared to control rats (Figs. 9 and 10). On the contrary, osteoarthritic rats receiving OA-F2L, M and H, showed up-regulation in the synovial expression of SOD by ~1.87-fold (*p* < 0.01), ~1.22-fold and ~1.29-fold, respectively compared to osteoarthritic control rats (Fig. 9). GPx expression was also elevated by ~2.51-fold (*p* < 0.01), ~1.36-fold and ~1.22-fold in osteoarthritic rats receiving OA-F2L, M and H, respectively (Fig. 9). Similarly, Up-regulation in the CAT expression by ~1.29-fold and ~1.15-fold, respectively was also observed in osteoarthritic rats receiving OA-F2M and H compared to osteoarthritic control rats (Fig. 10). OA-F2L did not show up-regulation in CAT expression. Rats receiving DICLO did not show any up-regulation in SOD, GPx or CAT expression. In the knee synovium, osteoarthritic control rats revealed up-regulation of MMP-3, MMP-9 and TIMP-1 by ~9.34-fold (*p* < 0.05), ~33.47-fold and ~1.57-fold when compared to control rats (Figs. 11 and 12). Significant down-regulation in expression of MMP-3 by ~4.45-fold (*p* < 0.05), ~8.55-fold (*p* < 0.01), ~3.58-fold (*p* < 0.05) and ~4.29-fold (*p* < 0.05) was observed in the osteoarthritic rats receiving OA-F2L, M, H and DICLO respectively (Fig. 11), compared to osteoarthritic control rats. These groups also showed down-regulation of MMP9 expression by ~7.85-fold, ~25.50-fold, ~19.38-fold and ~27.47-fold, respectively (Fig. 11). Up-regulation of TIMP-1 was observed in rats treated with OA-F2L, H and DICLO by ~1.52-fold, ~1.17-fold and ~1.02-fold, respectively when compared to osteoarthritic control rats (Fig. 12). OA-F2M was unable to modulate the level of synovial TIMP-1 in the osteoarthritic knee joints.

## Discussion

Osteoarthritis (OA) is a major cause of disability throughout the world, it causes pain due to inflamed knee joints, which involves progressive degeneration of articular cartilage, synovitis, formation of osteophyte, increased fibrillation due to increased denaturation and loss of collagen fibers [27]. Current therapeutic options for OA are mainly palliative and have little influence on progression of the disease and are also associated with many adverse effects [28]. Therefore, there is an increasing merit in use of compounds derived from natural plants or herbs for the treatment of OA [28]. In OA, cartilage degeneration is the result of combined biochemical and mechanical factors. Therefore, restoration of MMP-TIMP balance with the help of natural products that can effectively control oxidative stress and inflammation may provide an important therapeutic approach for the treatment of OA [29].

Intra-articular injection of collagenase significantly increased inflammation and swelling of the knee joint and thus increased diameter of the knee. Oral administration of OA-F2 significantly attenuated this inflammation and has reduced knee swelling and paw edema compared to osteoarthritic control group (Figs. 1 and 2). MMPs are the catalytic enzymes, which degrade the cartilage and in turn enhance the inflammatory process by releasing more cytokines and MMPs [27]. OA-F2 treatment to osteoarthritic rats showed significant improvement in paw withdrawal latency time compared to untreated osteoarthritic rats (Fig. 3). Serum CRP, probably the most widely used clinical marker of systemic inflammation, has been shown to correlate well with CRP in synovial fluid in patients with OA or RA [30]. In OA patients, elevated levels of CRP and ALP were recorded [25, 31]. Interestingly in the present study, CRP and ALP levels were significantly decreased by OA-F2 as compared to osteoarthritic control and diclofenac treated group (Figs. 4 and 5). Glycosaminoglycan is a major component of joint cartilage, joint fluid and other soft connective tissues. GAGs are released from the degrading cartilage matrix in a large amount. Administration of OA-F2, lowered GAG release in which means cartilage is protected as seen in the improved parameter of the joint space (Figs. 6 and 7), which was also confirmed in radiography. H and E staining showed that OA-F2 protected synovium and cartilage degeneration during OA (Fig. 8). Modification of ECM and increased articular chondrocyte proliferation are characteristics of OA [32], OA-F2 also protected chondroid matrix and prevented proteoglycan loss from ECM (Fig. 8). Histopathological observation suggests correlation of OA-F2 intervention with improved muscle degeneration, fibrillation, erosion of cartilage and chondroid matrix.

As observed in histopathology, studied biochemical and physiological parameters, deteriorating changes seen in OA induced rats are may be outcomes of elevated release of MMPs or these focal changes are might be due to secretion of pro-inflammatory cytokines with reactive oxygen species (ROS) from the inflamed synovium and from activated chondrocytes [27, 29]. ROS are also involved in the regulation of IL-1 effects mediated by NF-κB. In the progression of OA, NF-κB is a transcriptional factor that can be triggered by a number of stimuli, such as cytokines, excessive mechanical stress and degradation products of ECM [28]. Activated NF-κB regulates several catabolic enzymes involved in matrix degradation, including MMPs [28, 33]. MMP-3 has been shown to break down a number of ECM proteins, including fibronectin, laminin, denatured collagens and proteoglycans [28]. In addition to ECM degradation, MMP-3 is involved in the activation cascades of MMP-13 and gelatinases [28]. Since MMP-1 and MMP-3 play vital roles in ECM turnover, their regulation has been suggested to be useful therapeutic target [28]. On the other hand, TIMPs are the endogenous regulators of MMPs, which play important roles in maintaining homoeostasis with MMPs [28]. Imbalance between MMPs and TIMPs is a salient feature of OA progression, leading to disruption of the balance between ECM biosynthesis and degradation [28]. Therefore, exploration of the mechanisms through which the proteolytic activity, production and expression of MMPs are inhibited by plant-derived natural products may lead to new therapeutic strategies [34]. To determine whether OA-F2 may affect the destruction of articular cartilage, we examined the expression of the OA biomarkers MMP-3, MMP-9 and TIMP-1 in the synovium of collagenase-induced OA rats. Interestingly, our results demonstrated that the elevated MMP-3 and −9 expression in osteoarthritic control rats were decreased by OA-F2 supplementation. Present results demonstrated up-regulation of TIMP-1 by OA-F2L and OA-F2H in osteoarthritic rats. *S. cordifolia* extract was shown to inhibit COX enzyme and prostaglandin synthesis [11], whereas, *Z. officinale* decrease IL1, NO and PGE2 and LTB4 production as well as inhibit TNF-α production [11, 13, 14].

In summary, we evaluated the efficacy of orally administered OA-F2, a composition containing a blend of two medicinal plants, roots of *S. cordifolia* and rhizomes of *Z. officinale*, in collagenase induced OA model in rats. OA-F2 notably tackled the physiological problems and decreased levels of inflammatory markers such as CRP, ALP and GAG in the serum. We demonstrated at the mRNA level that OA-F2 inhibited the expression of MMP −3 and −9 and increased the expression of TIMP-1, all of which are classical biomarkers of inflammation and cartilage degradation in OA.

## Conclusions

Our study revealed chondroprotective effect of OA-F2, which is explained through modulation of biochemical parameters studied as well as expression of MMPs-TIMP genes in knee synovial tissue. OA-F2 thus merits consideration as an alternative therapy from natural sources for the treatment of OA. However, further preclinical and human clinical studies are necessary.

## Abbreviations

ALP: Alkaline phosphatase
AP-1: Activator protein-1
CAT: Catalase
COX: Cyclooxygenase
CRP: C-reactive protein
GAG: Glycosaminoglycan
GPx: Glutathione proxidase
IL-1: Interleukin-1
IL12: Interleukin 12
LTB4: Leukotriene B4
MMP: Matrix metalloproteinases
NF-κB: Nuclear factor kappa B
NO: Nitric oxide
OA: Osteoarthritis
PGE: Prostaglandin
ROS: Reactive oxygen species
SE: Standard error
SOD: Superoxide dismutase
TIMP: Tissue inhibitors of metalloproteinases
TNF-α: Tumor necrosis factor-α
